# A System Biology Approach Reveals New Targets for Human Thyroid Gland Toxicity in Embryos and Adult Individuals

**DOI:** 10.3390/metabo14040226

**Published:** 2024-04-16

**Authors:** Jeane Maria Oliveira, Jamilli Zenzeluk, Caroline Serrano-Nascimento, Marco Aurelio Romano, Renata Marino Romano

**Affiliations:** 1 Department of Medicine, Laboratory of Reproductive Toxicology, State University of the Midwest (UNICENTRO), Alameda Élio Antonio Dalla Vecchia, nº 838, Guarapuava 85040-167, PR, Brazil; 1712005@unicentro.edu.br (J.M.O.); 53021140002@unicentro.edu.br (J.Z.); mromano@unicentro.br (M.A.R.); 2 Institute of Environmental, Chemical and Pharmaceutical Sciences (ICAQF), Department of Biological Sciences, Federal University of São Paulo (UNIFESP), Rua Professor Arthur Riedel, 275, Diadema 09972-270, SP, Brazil; caroline.serrano@unifesp.br; 3 Department of Medicine, Laboratory of Molecular and Translational Endocrinology Medicine, Federal University of São Paulo (UNIFESP), Rua Pedro de Toledo, 669-11º andar-L11E, São Paulo 04039-032, SP, Brazil

**Keywords:** functional enrichment analysis, thyroid, development, toxicogenomics, thyroid toxicity

## Abstract

Compounds of natural or synthetic origin present in personal care products, food additives, and packaging may interfere with hormonal regulation and are called endocrine-disrupting chemicals (EDCs). The thyroid gland is an important target of these compounds. The objective of this study was to analyze public data on the human thyroid transcriptome and investigate potential new targets of EDCs in the embryonic and adult thyroid glands. We compared the public transcriptome data of adult and embryonic human thyroid glands and selected 100 up- or downregulated genes that were subsequently subjected to functional enrichment analysis. In the embryonic thyroid, the most highly expressed gene was PRMT6, which methylates arginine-4 of histone H2A (86.21%), and the downregulated clusters included plasma lipoprotein particles (39.24%) and endopeptidase inhibitory activity (24.05%). For the adult thyroid gland, the most highly expressed genes were related to the following categories: metallothionein-binding metals (56.67%), steroid hormone biosynthetic process (16.67%), and cellular response to vascular endothelial growth factor stimulus (6.67%). Several compounds ranging from antihypertensive drugs to enzyme inhibitors were identified as potentially harmful to thyroid gland development and adult function.

## 1. Introduction

The thyroid is a classical endocrine gland that is responsible for the production of thyroid hormones, which are essential for the regulation of energy metabolism, development, and growth. In addition, hormones produced by the thyroid regulate multiple physiological activities, including the cellular metabolic rate, cardiac and digestive functions, muscle function, brain development, and bone maintenance [1,2,3]. Many of the metabolic activities regulated by these hormones are related to the anabolism and/or catabolism of macromolecules, which affect energy homeostasis under different nutritional conditions [4].

Given the importance of the proper functioning of the hypothalamus–pituitary–thyroid axis for the maintenance of different functions in the body, the deregulation of this system negatively affects other organs and systems dependent on the actions of thyroid hormones [5,6]. In recent decades, epidemiological data have shown a high incidence and prevalence of diseases associated with exposure to endocrine-disrupting chemicals (EDCs), which are natural or synthetic compounds that interfere with the production, release, transport, metabolism, action, and/or secretion of endogenously produced hormones by the body [6,7,8,9,10,11]. These substances include pharmaceuticals, pesticides, personal care products, and surfactants, which are present in everyday life and whose concentrations are not commonly monitored in the environment [12]. Human exposure to these EDCs occurs via several routes, the most common of which are inhalation, ingestion, or dermal contact. Notably, most EDCs have lipophilic characteristics and therefore accumulate in adipose tissue, which makes co-contamination very frequent. In addition, lifetime exposure may have cumulative, additive, and/or synergistic effects [13].

EDCs do not show a classic toxicological pattern. Lower doses may induce more significant toxic effects than higher doses, thus hindering the determination of parameters such as the lowest-observed-adverse-effect level (LOAEL) and no-observed-adverse-effect level (NOAEL) [2]. The complexity of the effects induced by EDCs includes the “windows of vulnerability”. The developmental stage at which EDCs are exposed is crucial for affecting the endocrine system. Therefore, the embryonic and prepuberty periods are among the most susceptible to the deleterious effects promoted by exposure to EDCs [13,14]. Thus, the connection between the intrauterine life environment and the metabolic programming of individuals is directly related to the susceptibility of individuals to developing diseases in adulthood and supports the theory of the development origins of health and disease (DOHaD) [15,16,17]. The prepubertal phase, in turn, is an important period of development responsible for the profound biological, morphological, and cellular changes necessary for sexual reproduction in adulthood and is highly dependent on the action of hormones, which increases susceptibility to the deleterious effects triggered by exposure to EDCs [18]. Several EDCs deregulate thyroid function and the transport, bioavailability, action, and metabolism of thyroid hormones [8,19,20,21]. However, little is known about the impact of these substances on embryonic development and thyroid programming in adults.

Thus, the objective of this study was to evaluate, using system biology approaches, new targets of chemical agents with toxic potential in the thyroids of embryos and adult individuals. For this purpose, public human thyroid transcriptome data from both embryonic and adult life were used, and the gene expression profiles in these two periods were compared. The biological processes associated with the most highly expressed proteins in each period were determined. The possible chemical interactions between the proteins involved in these biological processes and chemical substances with potential thyroid toxicity were investigated. Based on this approach, new targets for possible thyroid toxicity caused by exposure to chemical agents were identified.

## 2. Materials and Methods

### 2.1. Dataset Selection

To evaluate the susceptibility of the thyroid gland to chemical compounds in general, we performed a study with bioinformatics tools using data from the literature on embryonic and adult thyroid transcriptomes [data available under Creative Commons license—[22] and GSE165706 (https://www.ncbi.nlm.nih.gov/geo (accessed on 4 February 2023) [23]). To obtain the transcriptomes, the RNA from embryonic (gestation weeks 7 to 11) and adult thyroids was hybridized onto Affymetrix arrays and compared to other human tissues of various organs from either fetuses or adults. For this study, we used the results of genes exclusively expressed in the thyroid. In this evaluation, we selected 100 genes whose transcripts increased in the embryonic thyroid, 100 whose transcripts increased in the adult thyroid, 100 whose transcripts decreased in the embryonic thyroid, and 100 whose transcripts decreased in the adult thyroid by evaluating the logFC between adult thyroids (ATs) and embryonic thyroids (ETs) [22] (Supplementary File S1).

### 2.2. Gene Set Enrichment Analysis

These genes were subjected to system biology analysis through the formation of a functional network using Cytoscape 3.9.1 software [24] and ClueGO v2.5.9 + CluePedia v1.5.9 [25,26]. The enrichment analysis by ClueGo was performed using the 4 categories of gene ontology (GO), namely biological processes, cellular components, immune system processes, and molecular function, and 2 categories of Reactome (pathways and reactions) and WikiPathways. The enriched networks were obtained from the following parameters: (1) mode of analysis: functional analysis; (2) list of charge markers: *Homo sapiens*; (3) visual style: groups; (4) ClueGO configurations: biological process + cellular components + molecular function; (5) network specificity: average; and (6) kappa score = 0.4 (standard).

### 2.3. Chemical Interaction Discovery

First, the genes related to the three most relevant biological processes were investigated for possible chemical interactions [27]. For each gene–chemical interaction, curated data were retrieved from the Comparative Toxicogenomics Database (CTD), MDI Biological Laboratory, Salisbury Cove, Maine, and NC State University, Raleigh, North Carolina (worldwide web URL: http://ctdbase.org/ (accessed on 4 February 2023)) [28]. On the home page, the link “What chemicals interact with a gene/protein?” was selected, and the “Chemical–Gene Interaction Query” was filled with the gene name (equals), selecting a gene from the list, and gene form (protein), and then we clicked search. The interacting chemicals are presented in the first column of the page.

Next, we sought to identify which of these chemical compounds had already been identified as thyrotoxic using the PubMed (https://pubmed.ncbi.nlm.nih.gov/ (accessed on 4 February 2023)) [29] search with the terms “name of the chemical”, “thyroid”, and “thyroid toxicity”. Those with no reported thyroid toxicity were classified as having new potential causes of toxicity. These chemical compounds were then classified according to the information available in PubChem (https://pubchem.ncbi.nlm.nih.gov/ (accessed on 4 February 2023)) [30].

## 3. Results

### 3.1. The Thyroid Embryonic Development Period as a Target for EDCs—A System Biology Approach

**Figure 1:**
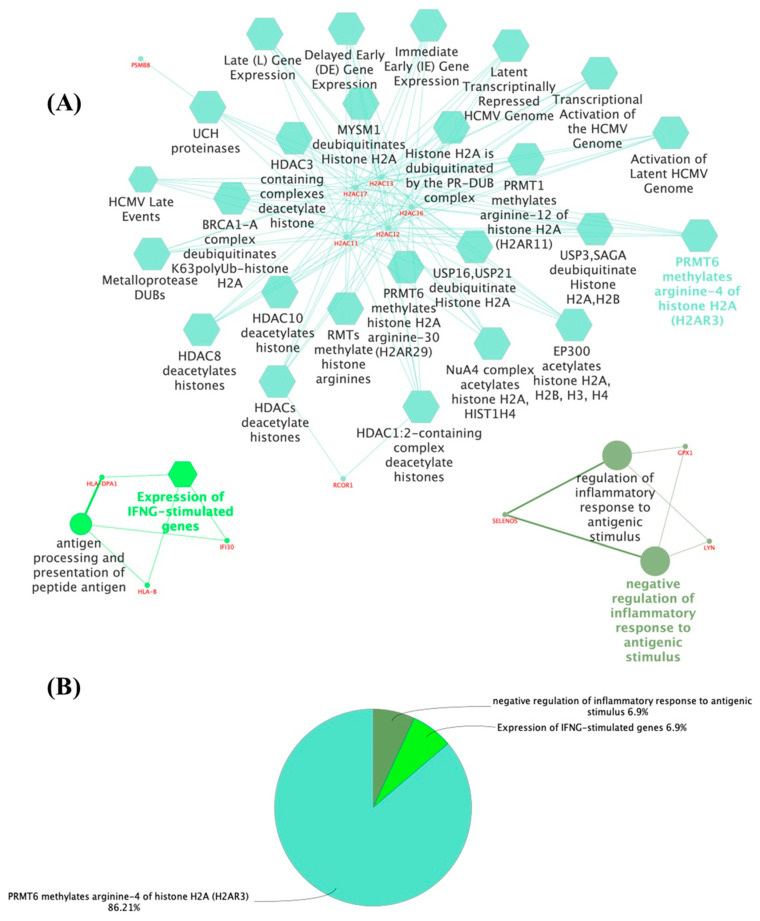
(**A**) Network of 100 proteins identified in the upregulated group of the embryonic thyroid according to functional enrichment analysis of ClueGo + CluePedia. The biological terms are differentially represented by colors and shapes, the sizes vary according to significance, and the most significant term is highlighted with the name of each group. The edges connect small nodes that represent related genes to the corresponding terms. Ontology used: GO_Biological Process; GO_Cellular Component; GO_Immune System Process; GO_Molecular Function (all GO terms are represented by circles); REACTOME_Pathways; REACTOME_Reactions (REACTOME terms are represented by hexagons); WikiPathways (not shown). (**B**) Pie chart of the enriched biological functions showing the percentage of the main terms generated from the proteins of the upregulated group of the embryonic thyroid.

The functional enrichment analysis of the embryonic and adult thyroid transcriptomes revealed new genes and proteins that may play a role in thyroid function. The proteins identified in each comparison were arranged in a functional network according to the most significant terms and showed different expression profiles. In the embryonic thyroid, the genes whose expression increased (Figure 1A,B; Supplementary File S2) included PRMT6, which methylates arginine-4 of histone H2A (H2AR3) (86.21%) (R-HSA:5205820) and IFNG-stimulated genes (6.9%) (R-HSA:1031716). Conversely, there was a downregulation of the inflammatory response to antigenic stimulus (6.9%) (GO:0002862). The most prominent cluster was the methylation of histones H2AC11, H2AC12, H2AC13, H2AC16, and H2AC17; interferon-gamma signaling proteins (HLA-B, HLA-DPA1, and IFI30); and the negative regulation of the acute inflammatory response to antigenic stimuli (GPX1, LYN, and SELENOS).

**Figure 2:**
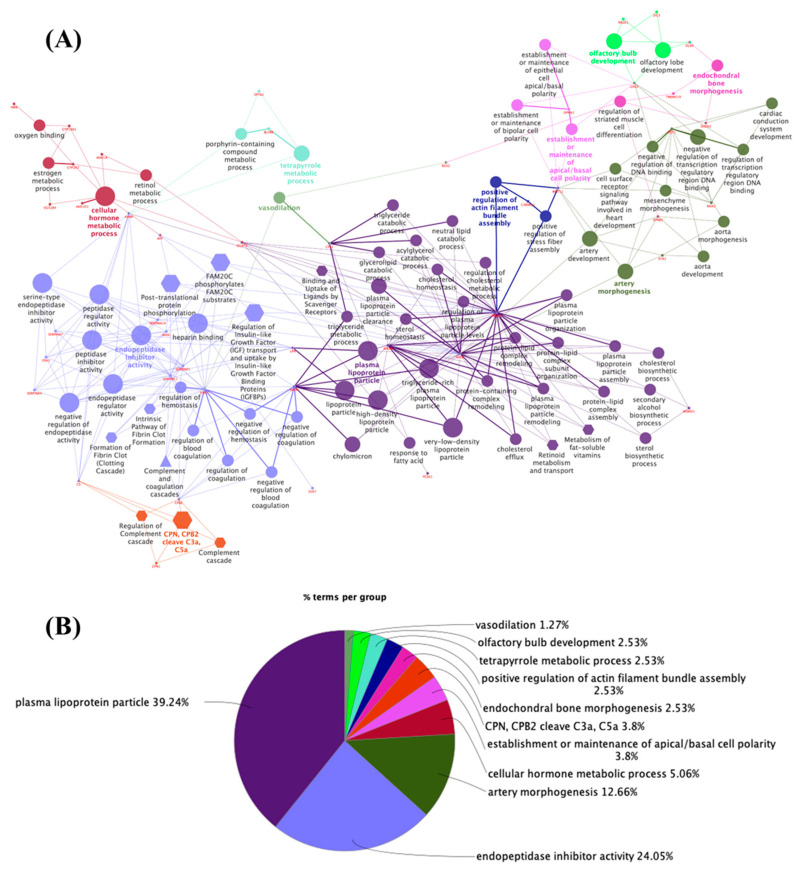
(**A**) Network of 100 proteins identified in the embryonic thyroid downregulation group, as determined by functional enrichment analysis of ClueGo + CluePedia. The biological terms are differentially represented by colors and shapes, the sizes vary according to significance, and the most significant term is highlighted with the name of each group. The edges connect small nodes that represent related genes to the corresponding terms. Ontology used: GO_Biological Process; GO_Cellular Component; GO_Immune System Process; GO_Molecular Function (all GO terms are represented by circles); REACTOME_Pathways; REACTOME_Reactions (REACTOME terms are represented by hexagons); WikiPathways (represented by triangles). (**B**) Pie chart of enriched biological functions showing the percentage of the main terms associated with the proteins in the downregulated embryonic thyroid group.

In the group of genes whose expression decreased in the embryonic thyroid (Figure 2A,B; Supplementary File S3), the enriched functions were plasma lipoprotein particle (39.24%; GO:0034358), endopeptidase inhibitor activity (24.05%; GO:0004866), artery morphogenesis (12.66%; GO:0048844), cellular hormone metabolic process (5.06%; GO:0034754), the establishment or maintenance of apical/basal cell polarity (3.8%; GO:0035088), ANC, CPB2 cleave C3a, C5a (3.8%; R-HSA:8852809), endochondral bone morphogenesis (2.53%; GO:0060350), the positive regulation of actin filament bundle assembly (2.53%; GO:0032233), tetrapyrrole metabolic process (2.53%; GO:0033013), olfactory bulb development (2.53%; GO:0021772), and vasodilation (1.27%; GO:0042311). The presented terms were related to apolipoproteins (APOA1, APOB, APOH, APOM, and LPA), glycoproteins (AHSG, AMBP, C5, ITIH1, KNG1, LPA, SERPINA10, SERPINA4, SERPINA7, SERPINC1, and SERPIND1), and proteins linked to morphogenesis (APOB, EFNB2, EYA1, HEY1, and WNT11).

Supplementary Tables S1 and S2 show the main compounds that potentially interact with proteins encoded by the genes that were differentially expressed in the embryonic thyroid. There are a large number of commonly used drugs with potential effects on the developing gland, such as antineoplastic agents (tamoxifen and triptolide), antioxidants (resveratrol, selenic acid, ascorbic acid, and isoquercitrin), anti-inflammatory agents (nimesulide and methylprednisolone), antihypertensive drugs (atenolol and valsartan), and natural compounds such as ginsenoside Rf and soybean oil.

### 3.2. Potential Targets for EDCs in the Adult Thyroid Gland—A System Biology Approach

**Figure 3:**
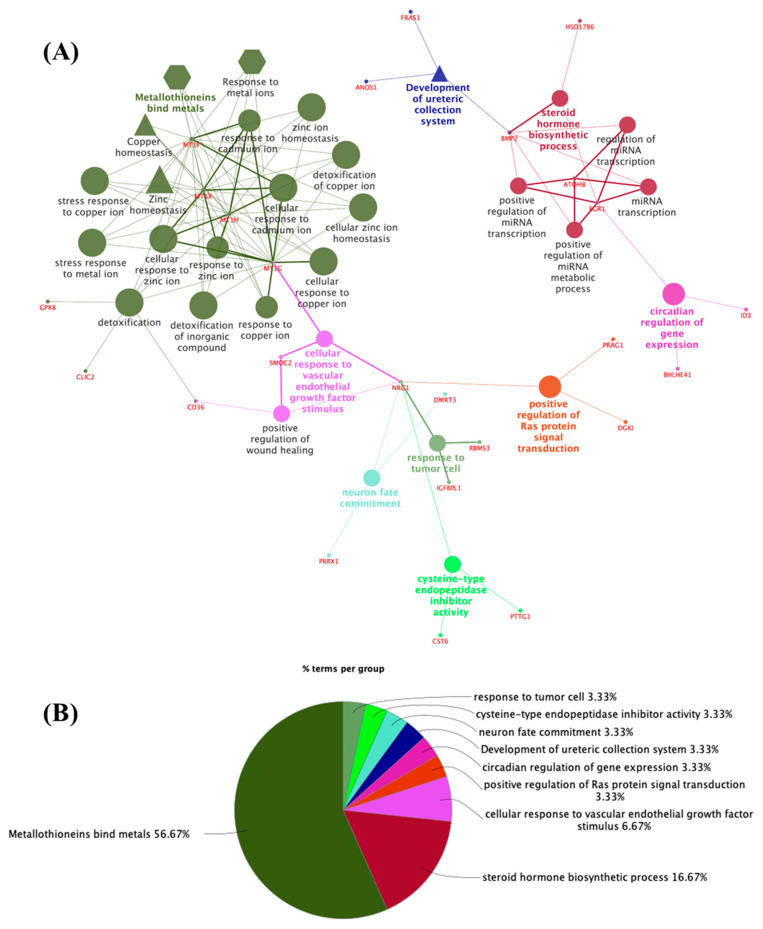
(**A**) Network of 100 proteins identified in the adult thyroid upregulation group, as determined by functional enrichment analysis of ClueGo + CluePedia. The biological terms are differentially represented by colors and shapes, the sizes vary according to significance, and the most significant term is highlighted with the name of each group. The edges connect small nodes that represent related genes to the corresponding terms. Ontology used: GO_Biological Process; GO_Cellular Component; GO_Immune System Process; GO_Molecular Function (all GO terms are represented by circles); REACTOME_Pathways; REACTOME_Reactions (REACTOME terms are represented by hexagons); WikiPathways (represented by triangles). (**B**) Pie chart of enriched biological functions showing the percentage of the main terms associated with the proteins in the adult thyroid upregulation group.

The network generated from 100 proteins encoded by increased genes in the adult thyroid (Figure 3A,B; Supplementary File S4) revealed a significant enrichment of the functions of metallothionein-binding metals (56.67%; R-HSA: 5661231), steroid hormone biosynthetic processes (16.67%; GO:0120178), cellular response to vascular endothelial growth factor stimulus (6.67%; GO:0035924), the positive regulation of Ras protein signal transduction (3.33%; GO:0046579), the circadian regulation of gene expression (3.33%; GO:0032922), ureteric collection system (3.33%; WP:5053), neuron fate commitment (3.33%; GO:0048663), cysteine-type endopeptidase inhibitor activity (3.33%; GO:0004869) and response to tumor cells (3.33%; GO:0002347). This analysis revealed that clusters related mainly to metal ion response/binding proteins (MT1F, MT1G, MT1H, and MT1X); proteins related to the transcriptional regulation of steroid hormones such as progesterone, aldosterone, and cortisol (BMP2, EGR1, and HSD17B6); and proteins involved in endothelial growth factor stimulation (MT1G, NRG1, and SMOC2).

**Figure 4:**
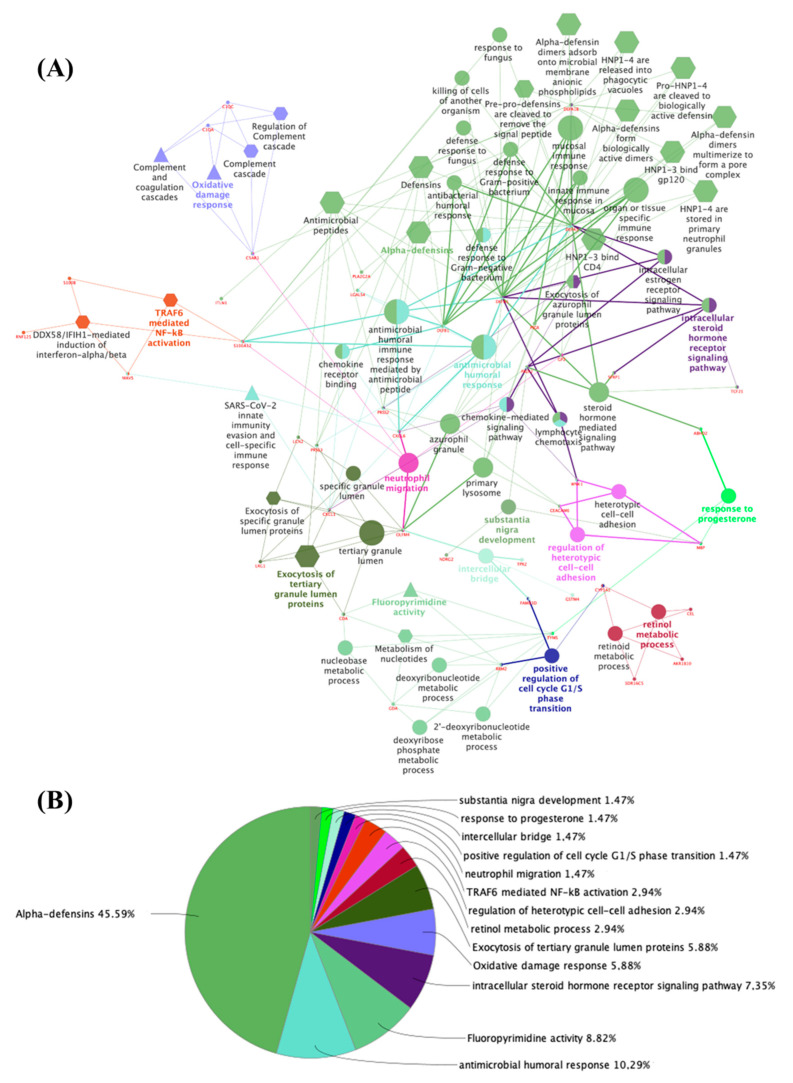
(**A**) Network of 100 proteins identified in the downregulated group of the adult thyroid according to functional enrichment analysis of ClueGo + CluePedia. The biological terms are differentially represented by colors and shapes, the sizes vary according to significance, and the most significant term is highlighted with the name of each group. The edges connect small nodes that represent related genes to the corresponding terms. Ontology used: GO_Biological Process; GO_Cellular Component; GO_Immune System Process; GO_Molecular Function (all GO terms are represented by circles); REACTOME_Pathways; REACTOME_Reactions (REACTOME terms are represented by hexagons); WikiPathways (represented by triangles). (**B**) Pie chart of enriched biological functions showing the percentage of the main terms associated with the proteins in the adult thyroid downregulation group.

Regarding the genes in the group whose expression decreased specifically in the adult thyroid in comparison to the embryonic tissue (Figure 4A,B; Supplementary File S5), the enriched terms were alpha-defensins (45.59%; R-HSA: 1462054), antimicrobial humoral response (10.29%; GO:0019730), fluoropyrimidine activity (8.82%; WP:1601), intracellular steroid hormone receptor signaling pathway (7.35%; GO:0030518), oxidative damage response (5.88%; WP:3941), the exocytosis of tertiary granule lumen proteins (5.88%; R-HSA:6798745), the retinol metabolic process (2.94%; GO:0042572), the regulation of heterotypic cell–cell adhesion (2.94%; GO:0034114), TRAF6-mediated NF-kB activation (2.94%; R-HSA:933542), neutrophil migration (1.47%; GO:1990266), the positive regulation of cell cycle G1/S phase transition (1.47%; GO:1902808), intercellular bridge (1.47%; GO:0045171), response to progesterone (1.47%; GO:0032570), and substantia nigra development (1.47%; GO:0021762).The main functions were related to defensin proteins (DEFA1, DEFA1B, DEFA3, PRSS2, and PRSS3); the chemokines CXCL1, CXCL6, DEFA1, DEFA3, DEFB1, LGALS4, PRSS2, PRSS3, and S100A12; proteins related to catalytic enzymes (CDA, RRM2, and TYMS); and steroid hormone signaling proteins (DEFA1, DEFA3, PADI2, SFRP1, and TCF21).

Supplementary Tables S3 and S4 show the main compounds that potentially interact with proteins differentially expressed in the adult thyroid gland. Therefore, these proteins may interact with antineoplastic agents (fenretinide, silybin, tretinoin, neocuproine, and triptolide), antihypertensive drugs (losartan, reserpine, and enalapril), enzyme inhibitors (propargylglycine, decitabine, and digoxin), nonsteroidal anti-inflammatory drugs (acetovanillone, mesalamine, and indomethacin) and enzyme inhibitors (benazepril, cyclosporine, and nolatrexed).

## 4. Discussion

The thyroid gland is a known target for EDCs [8,19,20,21], and little is known about the impact of these substances on embryonic development and thyroid programming in adults. For this reason, we identified new potential targets for thyroid toxicity using public embryonic and adult thyroid transcriptome data. Functional enrichment analysis revealed new genes and proteins that may play a role in thyroid function during development and adulthood.

The main function of the thyroid is to produce the hormones triiodothyronine (T3) and thyroxine (T4). Their synthesis and secretion are controlled by the hypothalamus–pituitary–thyroid (HPT) axis [3]. The HPT axis is a classic example of how a neuroendocrine system regulates the distinct functions of an organism during development and in adulthood in response to a variety of challenges [31].

The thyroid gland originates from endoderm embryonic tissue, and its development is dependent on the programmed and sequential expression of the homeobox transcription factor gene Hex (HHEX), thyroid transcription factor 1 (NKX2.1), thyroid transcription factor 2 (FOXE-1), and paired box gene 8 (PAX8) [32,33]. The coordinated expression of these transcription factors is related to the specification, budding, and migration of the precursor tissue to the thyroid gland from the third week of gestation. Interestingly, there is a temporal and structural correlation between the synthesis of thyroid hormones and folliculogenesis, suggesting that the structural and functional maturation of this gland are correlated [34]. The coordinated and sequential expression of the thyroid transcription factors culminates in the expression of thyroglobulin (TG), thyrotropin receptor (TSHR), and thyroperoxidase (TPO) around the 7th week of gestation. The expression of the sodium/iodide cotransporter (NIS), which is essential for iodide uptake and limits the synthesis of the thyroid hormones triiodothyronine (T3) and thyroxine (T4), occurs between the 8th and 9th gestational weeks [35].

In our study, among the genes whose expression increased in the embryonic thyroid, the most prominent cluster was associated with the methylation of histones H2AC11, H2AC12, H2AC13, H2AC16, and H2AC17. Indeed, covalent posttranslational modifications of the arginine termini of histones, such as methylation, acetylation, or phosphorylation, determine the state of active or repressed chromatin and play key roles in the regulation of gene expression and transcriptional memory during cell division; these modifications are finely controlled during the embryonic period [36].

Another related cluster upregulated in the thyroid embryonic tissue was the interferon-gamma signaling proteins HLA-B, HLA-DPA1, and IFI30. The MHC class is found in embryonic tissues [37] and seems to modulate cell proliferation by modulating the immune response via epigenetic mechanisms [38]. Interferon-gamma-inducible protein 30 (IFI30) is highly expressed in the caudal vein plexus (CVP) region of zebrafish embryos and is required for sprouting angiogenesis during structural development [39]. Interestingly, there is a possible association between HLA typing and congenital hypothyroidism. An association between an increased frequency of HLA-Aw24 was observed in a study from Japan [40], a case report of insulin-dependent diabetes mellitus with congenital myasthenia gravis and autoimmune hypothyroidism in a family with HLA-A29, B7, and DR6 haplotypes [41], and a case report of monozygotic twins discordant for Wiedemann–Beckwith syndrome (WBS) associated with congenital central hypothyroidism [42]. However, it was not identified in white American studies [43] or Danish studies [44,45].

Functional enrichment also revealed the proteins involved in the negative regulation of the acute inflammatory response to antigenic stimuli (GPX1, LYN, and SELENOS). The selenium incorporated into selenoproteins such as glutathione peroxidase 1 (GPx1) acts as a trace element for the biosynthesis of enzymes involved in antioxidant defense in follicular cells [46], modifying the redox status and thyroid hormone metabolism. During thyroid hormone synthesis, GPX1, GPX3, and TR1 are upregulated, providing thyrocytes with protection against peroxidative damage [47]. A deficiency of selenoproteins in pregnant mice reduces blood glucose and fetal weight, increases the concentrations of maternal thyroid hormones, and impairs the placental metabolism of thyroid hormones and the expression of placental nutrient transporters [48]. In humans, lower selenium concentrations during pregnancy may be associated with lower thyroid function and lower birth weight [49]. Therefore, this enrichment in antioxidant enzymes reinforces the importance of these proteins in embryonic thyroid development.

For main clusters that presented downregulated expression in the embryonic thyroid tissue were related to apolipoproteins (APOA1, APOB, APOH, APOM, and LPA), glycoproteins (AHSG, AMBP, C5, ITIH1, KNG1, LPA, SERPINA10, SERPINA4, SERPINA7, SERPINC1, and SERPIND1), and proteins linked to morphogenesis (APOB, EFNB2, EYA1, HEY1, and WNT11).

The influence of thyroid hormones on the hepatic production of lipoproteins is well known, and a reduction in their levels leads to dyslipidemia. However, there is still no information in the literature on the importance of these proteins, specifically in thyroid gland development [50]. Glycoproteins have several functions, including transporting hormones in the blood and functioning in cell-to-cell adhesion, and the carbohydrate portion of glycoproteins is often the key factor in cellular recognition [51]. Among these glycoproteins, SERPINA7, which encodes thyroxine-binding globulin (TBG), the most important thyroid transporter in the blood, stands out. However, there is no description of the regulatory effects of this gene on the thyroid gland itself. Among the proteins linked to morphogenesis, EYA1, which participates in the morphogenesis of organs connected to the third pharyngeal pouch (thymus, parathyroid, and thyroid), stands out. Interestingly, another member of this family, EYA3, participates in the regulation of TSH production as a function of photoperiod variations through the formation of an EYA3-SIX1-TEF complex that stimulates the expression of TSHB in the pituitary [52]. It is possible that other proteins of this family still not yet considered may play relevant roles in thyroid development and function.

As pointed out in the Results section, in the adult thyroid, the main cluster revealed for increased genes was related to metal ion response/binding proteins, such as MT1F, MT1G, MT1H, and MT1X, which are involved in numerous cellular processes, such as the binding and transport of zinc and copper ions, differentiation, proliferation, and apoptosis. In fact, metallothioneins seem to participate in heavy metal detoxification, zinc storage and transport, and redox biochemistry. The increased synthesis and expression of these proteins are linked to the papillary thyroid cancer lineage [53,54].

Proteins such as BMP2, EGR-1, and HSD17B6, which are involved in the transcriptional regulation of steroid hormones such as progesterone, aldosterone, and cortisol, and the MT1G, NRG1, and SMOC2 proteins involved in endothelial growth factor stimulation were also functionally enriched in the adult thyroid tissue.

The main function of BMPs, which are transforming growth factor β (TGFβ) superfamily members, is to stimulate osteoblastogenesis and bone mineralization [55,56]. Additionally, BMP is involved in cell growth, apoptosis, differentiation, and cell patterning and specification in numerous tissues. In the thyroid, BMP modulates TSH-induced thyrocyte function and proliferation [55]. In euthyroid men, bone mineral density and serum TSH concentration are positively correlated. In addition, BMP2 activity increases with increasing TSH concentration [57]. The EGR-1 gene belongs to a group of early-response genes (Cys2-His2-type zinc-finger transcription factors) that mediate growth, proliferation, differentiation, and apoptosis by stimulating many environmental signals, including growth factors, hormones, and neurotransmitters [58]. It is worth noting that *Egr1* mRNA expression was downregulated by TSH in canine thyroid cells [59]. The protein encoded by the HSD17B6 gene is involved in androgen catabolism, converting 3 alpha-diol to dihydrotestosterone and androsterone to epi-androsterone [60]. Polymorphisms of this gene are associated with polycystic ovary syndrome [61,62,63]. However, despite the gene being expressed in the thyroid [64], its function in the gland has not yet been elucidated.

MT1G modulates the cellular response to metal ions, such as zinc and cadmium [65]; protects cells against toxic metal ions or reactive oxygen species [66]; and is also involved in the cellular response to vascular endothelial growth factor stimuli [65]. In the thyroid, as previously discussed, metallothioneins are upregulated in follicular thyroid carcinoma and are a marker of thyroid stress in Graves’ disease [66]. The protein encoded by the NRG1 gene is a membrane glycoprotein that mediates cell–cell signaling and plays a critical role in the growth and development of multiple organ systems [67]. NRG1 was identified as a serum biomarker of papillary thyroid cancer [67]. In addition, in normal individuals, serum NRG1 levels are correlated with FT4 levels, suggesting a possible role for NRG1 in thyroid function [68,69]. SMOC2 is a member of the SPARC family of matricellular proteins that promotes matrix assembly and can stimulate endothelial cell proliferation and migration, as well as angiogenic activity [70]. Its expression is downregulated in papillary thyroid carcinomas, and SMOC2 positivity is associated with a better prognosis [71]. SMOC2 polymorphisms may also be associated with an increased risk for Hashimoto thyroiditis and Graves’ disease [72].

As described in the Results section, the main clusters that were downregulated in the adult thyroid tissue were related to defensin proteins (DEFA1, DEFA1B, DEFA3, PRSS2, and PRSS3); the chemokines (CXCL1, CXCL6, DEFA1, DEFA3, DEFB1, LGALS4, PRSS2, PRSS3, and S100A12); proteins related to catalytic enzymes (CDA, RRM2, and TYMS); and steroid hormone signaling proteins (DEFA1, DEFA3, PADI2, SFRP1, and TCF21).

Defensins are a major family of host defense peptides expressed predominantly in epithelial cells and neutrophils. They are part of the innate immune system and play a significant role in host defense against various pathogens [73]. The alpha-defensins DEFA1, DEFA1B, and DEFA3 are overexpressed in orbital adipose tissue from patients with Grave’s ophthalmopathy [74]. PRRS2 and PRSS3 are human trypsins that may be involved in the immune system [75]. Until now, none of these proteins have known effects on thyroid function.

Taken together, it is possible to note that the enriched terms and respective proteins that were up- and downregulated in the embryonic period were mainly related to the development and normal function of the thyroid gland. In the adult thyroid, the imbalanced expression of the clusters of genes that were enriched is commonly related to the development of thyroid pathologies, such as cancer and autoimmune thyroiditis. These data reinforce the importance of these genes in the maintenance of normal thyroid function.

It is important to emphasize that comparisons between gene expression levels were carried out among the genes expressed exclusively in embryonic and adult thyroids. Other genes commonly expressed in other tissues at these stages of development were not included in this study. Furthermore, it is not feasible to discern differences in gene expression related to gender, as embryonic and adult thyroid tissues were derived from both sexes and were evaluated together to obtain the transcriptome data [22].

Finally, we performed a search for unknown thyroid toxicity-related chemicals that target the proteins enriched in the three most relevant biological processes. The complete list of chemicals is presented in Supplementary Tables S1–S4. While 35 genes of the top five MeSH pharmacological classifications associated with increased proteins in the embryonic thyroid were associated with chemical compounds, these compounds interact with only two specific proteins, GPX1 and LYN.

As discussed before, the GPX1 gene encodes the classical protein glutathione peroxidase, which catalyzes the reduction of hydrogen peroxide, protecting cells against oxidative stress [76]. Although hydrogen peroxide is most commonly associated with the harmful effect of oxidative stress, it participates in several important biological functions that have already been described, such as growth factor-mediated signal transduction, mitochondrial function, and the maintenance of thiol redox balance; therefore, by limiting H2O2 accumulation, glutathione peroxidases are also involved in modulating these processes [77,78]. GPX1 was identified as a direct target of p53 in hypoxia, DNA damage induced by ionizing radiation or adriamycin, or the inhibition of topoisomerase II by etoposide [76]. The LYN gene encodes a tyrosine kinase protein that may be involved in the immune system. It is associated with and regulated by the IL-2 receptor complex on B and T lymphocytes [79].

Among the antineoplastic agents, fenretinide is a synthetic retinoid agonist that specifically binds to the retinoic acid receptor (RAR) [80]. Both the retinoid x receptor (RXR) and RAR are heterodimer partners of thyroid receptors (TRs) and are regulators of T3-modulated gene expression [81]. It is worth noting that, among the antioxidant agents listed, there are no known endocrine effects. Among the anti-inflammatory agents listed, only methylprednisolone has a described endocrine effect, but there is no evidence of its effect on thyroid function.

Honokiol, the molecular constituent of *Magnolia officinalis Rehder et Wilson* or *Magnolia grandiflora* L., is a Chinese medicinal plant [82]. Its pharmacological effects include anti-inflammatory, antithrombotic, antiarrhythmic, antioxidative, central depressant, muscle relaxant, and anxiolytic effects [82]. In the endocrine system, honokiol activates the ERK/c-fos signaling pathway, increases GDNF levels [83], increases the mRNA expression of glucocorticoid receptors in the hippocampus [84], improves insulin resistance in type 2 diabetic db/db mice [85], and inhibits 5-alpha-reductase type 1, modulating testosterone levels in vitro [82]. Nevertheless, its effects on the thyroid gland have never been reported.

Among the classes of natural compounds, none of the listed chemicals have known functions in the endocrine system. Triptolide, which is extracted from the common Chinese herb *Tripterygium wilfordii*, disrupts spermatogenesis by targeting SPEM1, acting as a nonhormonal and reversible male contraceptive [86].

In the adult thyroid gland, the enriched proteins may interact with antineoplastic agents (fenretinide, silybin, tretinoin, neocuproine, and triptolide), antihypertensive drugs (losartan, reserpine, and enalapril), enzyme inhibitors (propargylglycine, decitabine, and digoxin), nonsteroidal anti-inflammatory drugs (acetovanillone, mesalamine, and indomethacin) and enzyme inhibitors (benazepril, cyclosporine, and nolatrexed). Importantly, besides those compounds that were previously discussed in embryonic thyroid results, there are no data on the effects of the other compounds in the development/function of the thyroid gland.

## 5. Conclusions

In this study, we identified new proteins that may play a role in thyroid gland development and function. In addition, these proteins may also be potential targets for treating thyroid gland toxicity. This is especially important since common chemical compounds, from antihypertensive drugs to enzyme inhibitors, were identified in the curated database targeting these proteins. These findings highlight a critical gap in our knowledge regarding the specific toxicities these substances may exert on thyroid function. Consequently, further research is crucial to establish a comprehensive understanding of these interactions and pave the way for the development of targeted treatment strategies and preventative measures.

## Data Availability

The data presented in this study are available in this article and Supplementary Materials.

## Supplementary Materials

The following supporting information can be downloaded at https://www.mdpi.com/article/10.3390/metabo14040226/s1, Supplementary Table S1: Summary of the interactions between chemical compounds and upregulated embryonic genes in the thyroid; Supplementary Table S2: Summary of the interactions between chemical compounds and downregulated genes in the embryonic thyroid; Supplementary Table S3: Summary of the interactions between chemical compounds and upregulated genes in the adult thyroid; Supplementary Table S4: Summary of the interactions between chemical compounds and downregulated genes in the adult thyroid; Supplementary File S1: List of 100 genes specifically up- or downregulated in embryonic and adult thyroid tissues; Supplementary File S2: Functional enrichment datasets for upregulated genes in the embryonic thyroid; Supplementary File S3: Functional enrichment datasets for downregulated genes in the embryonic thyroid; Supplementary File S4: Functional enrichment datasets for upregulated genes in the adult thyroid; Supplementary File S5: Functional enrichment datasets for downregulated genes in the adult thyroid.
