# Supplementary material for: A System Biology Approach Reveals New Targets for Human Thyroid Gland Toxicity in Embryos and Adult Individuals
Source: Metabolites. 2024 Apr 16;14(4):226. doi: 10.3390/metabo14040226 (PMC11052307; doi:10.3390/metabo14040226)
Supplement: Supplementary file 1 [file metabolites-14-00226-s001.zip › metabolites-2942682-SI/Suppl Table S1 _ UP ET_CTD.pdf]

Supplementary Table S1 - Summary of the interaction between chemical compounds and upregulated genes in embryonic thyroid

Rank	MeSH Pharmacological Classification	Chemical Compound	PubChem CID	Gene/Protein interaction
1	Antineoplastic Agents	Atrasentan	159594	GPX1
		Fenretinide	5288209	GPX1
		Prolinedithiocarbamate	105009	GPX1
		Tamoxifen	2733526	GPX1
		Triptolide	107985	GPX1
		Alvocidib	5287969	LYN
		Bortezomib	387447	LYN
		Honokiol	72303	LYN
		Ponatinib	24826799	LYN
		Masitinib	10074640	LYN
2	Antioxidants	2-tert-butylhydroquinone	16043	GPX1
		Acetovanillone/ apocynin	2214	GPX1
		Probucol	4912	GPX1
		Prolinedithiocarbamate	105009	GPX1
		Resveratrol	445154	GPX1
		Selenic acid	1089	GPX1
		Diosmin	5281613	GPX1
2	Anti-Inflammatory Agents	Acetovanillone/ apocynin	2214	GPX1
		Ketorolac tromethamine	84003	GPX1
		Nimesulide	4495	GPX1
		Taxifolin	439533	GPX1
		Methylprednisolone	6741	GPX1
		Thymoquinone	10281	GPX1
		Trilobatin	6451798	GPX1
4	Natural compound	Ginsenoside Re	53789984 (SID)	GPX1
		Isocytisoside	160749	GPX1
		Thymoquinone	10281	GPX1
		Triptonide	65411	LYN
		Trilobatin	6451798	GPX1
		Zingiberis rhizome carbonisata	442882585 (SID)	GPX1
5	Enzyme Inhibitors	2-tert-butylhydroquinone	16043	GPX1
		Acetovanillone/ apocynin	2214	GPX1
		Resveratrol	445154	GPX1
		Honokiol	72303	LYN
		Mycophenolic acid	446541	LYN
6	Indicators and Reagents	Phenolsulfonphthalein	4766	H2AC12
		Brucine	442021	GPX1
		Thioacetamide	2723949	GPX1
		Benzamide	2331	LYN
6	Protein Kinase Inhibitors	Alvocidib	5287969	LYN
		Bafetinib	11387605	LYN
		Masitinib	10074640	LYN
		Ponatinib	24826799	LYN
7	Antiparasitic/ Pediculicide	Ivermectin	6321424	H2AC12<br>HLA-B<br>GPX1<br>LYN
7	Herbicides	1-methyl-4-phenylpyridinium (Cyperquat)	39484	GPX1
		Arsenic acid	234	GPX1
		Diaminochlorotriazine	21897015	GPX1
7	Anti-Bacterial Agents	Ampicillin	6249	GPX1
		Metronidazole	4173	GPX1
		Vancomycin	14969	GPX1

7	Flavouring Agents	Carvacrol	10364	GPX1
		Diphenyl disulfide	13436	GPX1
		Raspberry ketone	21648	GPX1
7	dyes	Gallein	73685	GPX1
		Phycocyanin	53837743 (SID)	GPX1
		Benzanthrone	6697	LYN
7	Anti-Infective Agents	Metronidazole	4173	GPX1
		Furaltadone	9553856	LYN
		Honokiol	72303	LYN
8	Cholinesterase Inhibitors	Paraoxon	9395	H2AC12
		Phosphamidon	3032604	GPX1
8	Insecticides	Paraoxon	9395	H2AC12
		Phosphamidon	3032604	GPX1
8	carcinogenic agent	Monomethylarsonous acid	161491	HLA-B
		Pirinixic acid	5694	GPX1
8	Food additives	Allyl methyl sulfide	66282	GPX1
		Trehalose	7427	GPX1
8	Antirheumatic Agents	Auranofin	24199313	GPX1
		Thiomalates	136368256 (SID)	GPX1
8	Antibiotics, Antineoplastic	Bleomycin	5360373	GPX1
		Mycophenolic acid	446541	LYN
8	Peroxisome Proliferators	Ciprofibrate	2763	GPX1
		Pirinixic acid	5694	GPX1
8	Alkylating Agents	Diethylnitrosamine	5921	GPX1
		Triptolide	107985	GPX1
8	Cyclooxygenase Inhibitors	Ketorolac tromethamine	84003	GPX1
		Nimesulide	4495	GPX1
8	skin conditioning	Luteolin	5280445	GPX1
		Rhododendrol	919205	GPX1
8	Antibacterial	Neomycin	8378	GPX1
		Thymoquinone	10281	GPX1
8	Anticholesteremic Agents	Pirinixic acid	5694	GPX1
		Probucol	4912	GPX1
8	Trace Elements	Selenic acid	1089	GPX1
		Vanadates	61672	LYN
8	anti-oxidative	Thymoquinone	10281	GPX1
		Trilobatin	6451798	GPX1
8	Immunomodulatory/ Immunosuppressive Agents	Thymoquinone	10281	GPX1
		Triptolide	107985	GPX1
29	Coloring Agents	Phenolsulfonphthalein	4766	H2AC12
29	Anti-HIV Agents	Abacavir	441300	HLA-B
29	Reverse Transcriptase Inhibitors	Abacavir	441300	HLA-B
29	Flavoring Agents	Allyl methyl sulfide	66282	GPX1
29	Chemosterilants	Alpha-chlorohydrin	7290	GPX1
29	Teratogens	Arsenic acid	234	GPX1
29	Endothelin A Receptor Antagonists	Atrasentan	159594	GPX1
29	Adjuvants, Immunologic	Brucine	442021	GPX1
29	Analgesics	Brucine	442021	GPX1
29	Antimetabolites, Antineoplastic	Capecitabine	60953	GPX1
29	Hypolipidemic Agents	Ciprofibrate	2763	GPX1
29	Mast Cell Stabilizers	Cromolyn sodium	27503	GPX1
29	Anti-Asthmatic Agents	Cromolyn sodium	27503	GPX1
29	Oxidants	Cumene hydroperoxide	6629	GPX1
29	Mineralocorticoids	Desoxycorticosterone	6166	GPX1
29	Succinates	Dimethyl mercaptosuccinate	95335	GPX1
29	Cannabinoid Receptor Agonists	Dronabinol	16078	GPX1
29	Analgesics, Non-Narcotic	Dronabinol	16078	GPX1
29	Hallucinogens	Dronabinol	16078	GPX1
29	Psychotropic Drugs	Dronabinol	16078	GPX1
29	Anticarcinogenic Agents	Fenretinide	5288209	GPX1
29	Hematinics	Folic acid	135398658	GPX1

29	Vitamin B Complex	Folic acid	135398658	GPX1
29	Dopamine Agents	Levodopa	6047	GPX1
29	Antiparkinson Agents	Levodopa	6047	GPX1
29	Fungicides, Industrial	Maneb	3032581	GPX1
29	Central Nervous System Stimulants	Mephedrone	45266826	GPX1
29	Glucocorticoids	Methylprednisolone	6741	GPX1
29	Antiemetics	Methylprednisolone	6741	GPX1
29	Neuroprotective Agents	Methylprednisolone	6741	GPX1
29	Antiprotozoal Agents	Metronidazole	4173	GPX1
29	Aminoglycoside	Neomycin	8378	GPX1
29	Mutagens	Pirinixic acid	5694	GPX1
29	Platelet Aggregation Inhibitors	Resveratrol	445154	GPX1
29	Bone Density Conservation Agents	Tamoxifen	2733526	GPX1
29	Estrogen Antagonists	Tamoxifen	2733526	GPX1
29	Selective Estrogen Receptor Modulators	Tamoxifen	2733526	GPX1
29	Catalyst	Tert-butylhydroperoxide	6410	GPX1
29	Chelating Agents	Trientine	5565	GPX1
29	Antispermatogenic Agents	Triptolide	107985	GPX1
29	Antihypertensive Agents	Valsartan	60846	GPX1
29	Angiotensin II Type 1 Receptor Blockers	Valsartan	60846	GPX1
29	Mouthwashes	Zinc chloride	5727	GPX1
29	Cariostatic Agents	Aluminum fluoride	2124	LYN
29	Growth Inhibitors	Alvocidib	5287969	LYN
29	Anti-Anxiety Agents	Honokiol	72303	LYN
29	Anti-Arrhythmia Agents	Honokiol	72303	LYN
29	Anti-Allergic Agents	Honokiol	72303	LYN
29	Phytogenic	Honokiol	72303	LYN
29	Central Nervous System Depressants	Honokiol	72303	LYN
29	Gastrointestinal Agents	Honokiol	72303	LYN
29	Antibiotics, Antitubercular	Mycophenolic acid	446541	LYN
