# Supplementary material for: A System Biology Approach Reveals New Targets for Human Thyroid Gland Toxicity in Embryos and Adult Individuals
Source: Metabolites. 2024 Apr 16;14(4):226. doi: 10.3390/metabo14040226 (PMC11052307; doi:10.3390/metabo14040226)
Supplement: Supplementary file 1 [file metabolites-14-00226-s001.zip › metabolites-2942682-SI/Suppl Table S2 _ Down ET_CTD.pdf]

Supplementary Table S2 - Summary of the interaction between chemical compounds and downregulated genes in embryonic thyroid

Rank	MeSH Pharmacological Classification	Chemical Compound	PubChem CID	Gene/Protein interaction
1	Antihypertensive Agents	Atenolol	2249	APOA1<br>APOB
		Clonidine	2803	APOA1
		Benazepril	5362124	APOB<br>AMBP<br>KNG1
		Bendroflumethiazide	2315	APOB
		Linsidomine	5219	APOB
		Valsartan	60846	APOB<br>KNG1
		Diltiazem	39186	KNG1
		Enalapril	5388962	KNG1
		Perindopril	107807	KNG1
		Ramipril	5362129	KNG1
2	Antioxidants	Ascorbic acid	54670067	APOA1<br>APOB<br>C5
		Isoquercitrin	5280804	APOA1
		Probucol	4912	APOA1
		Resveratrol	445154	APOA1<br>APOB<br>APOM<br>AHSG<br>C5<br>SERPINC1
		Chrysin	5281607	APOB
		Gallic acid	370	APOB
		Ginger extract	459636550 (SID)	APOM
2	Natural compound	Coconut oil	350085943 (SID)	APOA1
		Ginsenoside Rf	441922	APOA1
		Perilla seed oil	488414420 (SID)	APOA1
		Rapeseed oil	350085953 (SID)	APOA1<br>APOB
		Safflower oil	481189677 (SID)	APOA1
		Soybean oil	53789874 (SID)	APOA1
		Chrysin	5281607	APOB
		Gallic acid	370	APOB
		Ginger extract	459636550 (SID)	APOM
		Erianin	356759	ITIH1
		Resiniferatoxin	5702546	KNG1
		Zingiberis rhizome carbonisata	442882585 (SID)	SERPINA7
2	Anti-Inflammatory Agents, Non-Steroidal	Sulindac sulfide	5352624	APOA1
		Taxifolin	439533	APOA1<br>APOB
		Indomethacin	3715	APOB<br>KNG1
		Sulindac	1548887	APOB
		Parthenolide	7251185	C5
		Flurbiprofen	3394	KNG1
		Icatibant	6918173	KNG1
		Meloxicam	54677470	KNG1

		Nafamostat	4413	KNG1
		Nimesulide	4495	KNG1
		Pyranoprofen	4888	KNG1
6	Enzyme Inhibitors	Cyclosporine	5284373	APOA1<br>SERPINC1
		Resveratrol	445154	APOA1<br>APOB<br>APOM<br>AHSG<br>C5<br>SERPINC1
		Linsidomine	5219	APOB
		Thapsigargin	446378	APOB<br>EYA1
		Pimagedine	2146	LPA
		Cyclosporine	5284373	APOA1<br>SERPINC1
6	Anticholesteremic Agents	Ezetimibe	150311	APOA1
		Lovastatin	53232	APOA1<br>APOB<br>LPA
		Pirinixic acid	5694	APOA1<br>APOB<br>APOH
		Probucol	4912	APOA1
		Rosuvastatin calcium	5282455	APOA1<br>APOB
9	Immunosuppressive Agents	Cyclosporine	5284373	APOA1<br>SERPINC1
		Fingolimod hydrochloride	107969	APOA1
		Triptolide	107985	APOA1<br>AMBP<br>KNG1<br>SERPINC1
		Busulfan	2478	SERPINC1
9	Antineoplastic agent	Ginsenoside Rf	441922	APOA1
		Pyrazolanthrone	8515	APOA1
		Sulindac sulfide	5352624	APOA1
		Chrysin	5281607	APOB
		Gallic acid	370	APOB
		Sulindac	1548887	APOB
		Tretinoin	444795	AMBP
9	Hypolipidemic Agents	Bezafibrate	39042	APOA1<br>APOB
		Ciprofibrate	2763	APOA1
		Fenofibrate	3339	APOA1<br>APOB
		Gemfibrozil	3463	APOA1<br>APOB
9	Platelet Aggregation Inhibitors	Resveratrol	445154	APOA1<br>APOB<br>APOM<br>AHSG<br>C5<br>SERPINC1
		Linsidomine	5219	APOB
9	Anti-Bacterial Agents	Ampicillin	6249	AHSG
		Metronidazole	4173	AHSG
		Vancomycin	14969	AHSG
		Amphotericin B	5280965	AMBP
		Neomycin	8378	AHSG
		Octanoic acid	379	KNG1<br>SERPINA7
12	Angiotensin-Converting Enzyme Inhibitors	Benazepril	5362124	APOB<br>AMBP

				KNG1
		Enalapril	5388962	KNG1
		Perindopril	107807	KNG1
		Ramipril	5362129	KNG1
12	Antineoplastic Agents, Phytogenic	Senecionine	5280906	APOB<br>APOH<br>APOM<br>C5<br>SERPINA10<br>SERPINA7
14	Food Additives	Dioleoyl ethylene glycol	5378708	APOA1
		Potassium persulfate	24412	APOA1
		Sodium acetate	517045	APOB
14	Flame retardant	Hexabrominated diphenyl ether 153 (PBDE 153)	53787637 (SID)	APOA1<br>APOB<br>KNG1<br>SERPINA10<br>SERPINA7
14	Hydroxymethylglutaryl-CoA Reductase Inhibitors	Lovastatin	53232	APOA1<br>APOB<br>LPA
		Rosuvastatin calcium	5282455	APOA1<br>APOB
14	Antiemetics	Olanzapine	135398745	APOA1<br>APOB<br>APOH<br>C5<br>KNG1
14	Antipsychotic Agents	Olanzapine	135398745	APOA1<br>APOB<br>APOH<br>C5<br>KNG1
14	Selective Serotonin Reuptake Inhibitors	Olanzapine	135398745	APOA1<br>APOB<br>APOH<br>C5<br>KNG1
14	Antineoplastic Agents, Alkylating	Triptolide	107985	APOA1<br>AMBP<br>KNG1<br>SERPINC1
		Busulfan	2478	SERPINC1
14	Cyclooxygenase Inhibitors	Indomethacin	3715	APOB<br>KNG1
		Sulindac	1548887	APOB
		Flurbiprofen	3394	KNG1
		Nimesulide	4495	KNG1
22	Anti-Arrhythmia Agents	Atenolol	2249	APOA1<br>APOB
		Losartan	3961	AMBP<br>KNG1
22	Peroxisome Proliferators	Ciprofibrate	2763	APOA1
		Pirinixic acid	5694	APOA1<br>APOB<br>APOH
22	Antifungal Agents	Amphotericin B	5280965	AMBP
		Cyclosporine	5284373	APOA1<br>SERPINC1
		Cycloheximide	6197	KNG1
22	Solvents	Dimethyl sulfoxide	679	APOA1
		Trichloroethylene (TCE)	6575	AMBP<br>C5<br>KNG1
22	Hypoglycemic Agents	Glyburide	3488	APOA1
		Pioglitazone	4829	APOA1

				APOB
		Phenformin	8249	SERPINC1
22	Carcinogens	Pirinixic acid	5694	APOA1<br>APOB<br>APOH
		Aristolochic acid I	2236	C5
22	Mutagens	Pirinixic acid	5694	APOA1<br>APOB<br>APOH
		Aristolochic acid I	2236	C5
22	Antispermatogenic Agents	Triptolide	107985	APOA1<br>AMBP<br>KNG1<br>SERPINC1
22	Gout Suppressants	Indomethacin	3715	APOB<br>KNG1
		Colchicine	6167	AMBP
		Pyranoprofen	4888	KNG1
22	Angiotensin II Type 1 Receptor Blockers	Valsartan	60846	APOB<br>KNG1
		Losartan	3961	AMBP<br>KNG1
32	Sympatholytics	Atenolol	2249	APOA1<br>APOB
		Clonidine	2803	APOA1
32	Vasodilator Agents	Bucladesine	9687	APOA1
		Linsidomine	5219	APOB
		Diltiazem	39186	KNG1
32	Analgesics	Clonidine	2803	APOA1
		Flurbiprofen	3394	KNG1
		Ketamine	3821	KNG1
32	Antirheumatic Agents	Cyclosporine	5284373	APOA1<br>SERPINC1
		Glucosamine	53786989 (SID)	APOB
32	Dermatologic Agents	Cyclosporine	5284373	APOA1<br>SERPINC1
		Hyaluronic acid	481108865 (SID)	ITIH1
32	Alkylating Agents	Diethylnitrosamine	5921	APOA1
		Busulfan	2478	SERPINC1
		Mitomycin	5746	HEY1
32	Antipruritics	Menthol	1254	APOA1
		Capsaicin	1548943	APOB
32	Tocolytic Agents	Sulindac sulfide	5352624	APOA1
		Indomethacin	3715	APOB<br>KNG1
32	Reagents	Ethanolamine	700	APOB
		Thioacetamide	2723949	APOB
		Potassium chloride	4873	KNG1
32	Cardiovascular Agents	Indomethacin	3715	APOB<br>KNG1
		Diltiazem	39186	KNG1
32	Antiparasitic/ Pediculicide	Ivermectin	6321424	APOB<br>C5<br>SERPINC1
32	Protease Inhibitors	Isoflurophate	5936	APOH
		Nafamostat	4413	KNG1
		Racecadotril	107751	KNG1
32	Estrogen Antagonists	Danazol	28417	LPA
		Tamoxifen	2733526	SERPINA7<br>SERPINC1
32	Bone Density Conservation Agents	Zoledronic acid	68740	AHSG
		Tamoxifen	2733526	SERPINA7<br>SERPINC1

32	Anesthetics, Inhalation	Trichloroethylene (TCE)	6575	AMBP<br>C5<br>KNG1
32	Anti-coagulant, antithrombotic properties	Heparin	481108841 (SID)	C5<br>SERPINC1
		Dalteparin	772	SERPINC1
48	Adrenergic beta-1 Receptor Antagonists	Atenolol	2249	APOA1<br>APOB
48	Calcineurin Inhibitors	Cyclosporine	5284373	APOA1<br>SERPINC1
48	Cytochrome P-450 CYP2C8 Inhibitors	Gemfibrozil	3463	APOA1<br>APOB
48	Chelating Agents	Pentetic acid	3053	APOA1
		Trientine	5565	APOA1
48	kinase inhibitor	Pyrazolanthrone	8515	APOA1
		Chrysin	5281607	APOB
48	Antiparkinson Agents	Tolcapone	4659569	APOA1<br>APOB
48	Catechol O-Methyltransferase Inhibitors	Tolcapone	4659569	APOA1<br>APOB
48	Sensory System Agents	Capsaicin	1548943	APOB
48	Anti-inflammatory agents	Chrysin	5281607	APOB
		Hydrocortisone	5754	SERPINC1
48	Adjuvants, Immunologic	Aconitine	245005	APOM<br>KNG1
48	Voltage-Gated Sodium Channel Agonists	Aconitine	245005	APOM<br>KNG1
48	Antibiotics, Antineoplastic	Bleomycin	5360373	AHSG
		Mitomycin	5746	HEY1
48	Antiprotozoal Agents	Metronidazole	4173	AHSG
		Amphotericin B	5280965	AMBP
48	Tubulin Modulators	Colchicine	6167	AMBP
		Oryzalin	29393	KNG1
48	Anticoagulants	Nafamostat	4413	KNG1
		Calcium heparin	134224540 (SID)	SERPINC1
48	Contraceptive Agents, Hormonal / Contraceptives, Oral, Synthetic	Norgestimate	6540478	SERPINA7<br>SERPINC1
48	Antineoplastic Agents, Hormonal	Tamoxifen	2733526	SERPINA7<br>SERPINC1
48	Selective Estrogen Receptor Modulators	Tamoxifen	2733526	SERPINA7<br>SERPINC1
66	antidiabetics	Apabetalone	135564749	APOA1
66	BET Inhibitor	Apabetalone	135564749	APOA1
66	Chemical Warfare Agents	Chlorine	24526	APOA1
66	Adrenergic alpha-2 Receptor Agonists	Clonidine	2803	APOA1
66	Cryoprotective Agents	Dimethyl sulfoxide	679	APOA1
66	Free Radical Scavengers	Dimethyl sulfoxide	679	APOA1
66	Sphingosine 1 Phosphate Receptor Modulators	Fingolimod hydrochloride	107969	APOA1
66	Anti-HIV Agents	Nevirapine	4463	APOA1
66	Cytochrome P-450 CYP3A Inducers	Nevirapine	4463	APOA1
66	Reverse Transcriptase Inhibitors	Nevirapine	4463	APOA1
66	Iron Chelating Agents	Pentetic acid	3053	APOA1
66	Antidotes	Pentetic acid	3053	APOA1
66	Anticarcinogenic Agents	Perilla seed oil	488414420 (SID)	APOA1
66	Antiseptic	Potassium persulfate	24412	APOA1
66	Diuretics	Bendroflumethiazide	2315	APOB
66	Sodium Chloride Symporter Inhibitors	Bendroflumethiazide	2315	APOB
66	hepatoprotective agent	Chrysin	5281607	APOB
66	Preservative	Diethyl pyrocarbonate	3051	APOB

66	Sclerosing Agent	Ethanolamine	700	APOB
66	Astringent	Gallic acid	370	APOB
66	HIV Protease Inhibitors	Indinavir	5362440	APOB
66	Nitric Oxide Donors	Linsidomine	5219	APOB
66	Muscarinic Antagonists	Propiverine	4942	APOB
66	Urological Agents	Propiverine	4942	APOB
66	Parasympatholytics	Propiverine	4942	APOB
66	Anti-Obesity Agents	Rimonabant	104850	APOB
66	Cannabinoid Receptor Antagonists	Rimonabant	104850	APOB
66	Antimetabolites, Antineoplastic	Gemcitabine	60750	APOH
66	Cholinesterase Inhibitors	Isoflurophate	5936	APOH
66	Anti-Infective Agents	Metronidazole	4173	AHSG
66	Amebicides	Amphotericin B	5280965	AMBP
66	Keratolytic Agents	Tretinoin	444795	AMBP
66	Siderophores	Deferoxamine	2973	C5
66	Salts	Dithionite	1086	C5
66	Laxative	Magnesium chloride	5360315	ITIH1
66	Uricosuric Agents	Benzbromarone	2333	KNG1
66	Protein Synthesis Inhibitors	Cycloheximide	6197	KNG1
66	Calcium Channel Blockers	Diltiazem	39186	KNG1
66	Oxytocics	Dinoprostone	5280360	KNG1
66	Bradykinin B2 Receptor Antagonists	Icatibant	6918173	KNG1
66	Anesthetics, Dissociative	Ketamine	3821	KNG1
66	Excitatory Amino Acid Antagonists	Ketamine	3821	KNG1
66	Cyclooxygenase 2 Inhibitors	Meloxicam	54677470	KNG1
66	Serine Proteinase Inhibitors	Nafamostat	4413	KNG1
66	Trypsin Inhibitors	Nafamostat	4413	KNG1
66	Complement Inactivating Agents	Nafamostat	4413	KNG1
66	Coccidiostats	Oryzalin	29393	KNG1
66	Herbicides	Oryzalin	29393	KNG1
66	Anesthetics, Intravenous	Propofol	4943	KNG1
66	Hypnotics and Sedatives	Propofol	4943	KNG1
66	Histamine H1 Antagonists	Pyrilamine	4992	KNG1
66	Anti-Allergic Agents	Pyrilamine	4992	KNG1
66	Sleep Aids, Pharmaceutical	Pyrilamine	4992	KNG1
66	Antidiarrheals	Racecadotril	107751	KNG1
66	Bronchodilator Agents	Verlukast	6509849	KNG1
66	Leukotriene Antagonists	Verlukast	6509849	KNG1
66	organobromine compound	Hexabromodiphenyl ether 154 (BDE 154)	15509898	SERPINA7
66	Antifibrinolytic Agents	Aminocaproic acid	564	SERPINC1
66	Myeloablative Agonists	Busulfan	2478	SERPINC1
66	Fibrinolytic Agents	Calcium heparin	134224540 (SID)	SERPINC1
66	Calcineurin Inhibitors	Cyclosporine	5284373	APOA1 SERPINC1
66	Cross-Linking Reagents	Mitomycin	5746	HEY1
66	Nucleic Acid Synthesis Inhibitors	Mitomycin	5746	HEY1
