# Supplementary material for: A System Biology Approach Reveals New Targets for Human Thyroid Gland Toxicity in Embryos and Adult Individuals
Source: Metabolites. 2024 Apr 16;14(4):226. doi: 10.3390/metabo14040226 (PMC11052307; doi:10.3390/metabo14040226)
Supplement: Supplementary file 1 [file metabolites-14-00226-s001.zip › metabolites-2942682-SI/Suppl Table S3 _ UP AT_CTD.pdf]

Supplementary Table S3 - Summary of the interaction between chemical compounds and upregulated genes in adult thyroid

Rank	MeSH Pharmacological Classification	Chemical Compound	PubChem CID	Gene/Protein interaction
1	Enzyme Inhibitors	Propargylglycine	185909	MT1G MT1X
		2-(4-nitrophenyl)-4-(4-fluorophenyl)-5-(4-pyridinyl)-1H-imidazole	4712	BMP2
		4-(4-fluorophenyl)-2-(4-hydroxyphenyl)-5-(4-pyridyl)imidazole	5169	BMP2 EGR1
		Decitabine	451668	BMP2
		Digoxin	2724385	EGR1
		Fluoranthene	9154	EGR1
		Resveratrol	445154	BMP2 EGR1 NRG1
2	Antineoplastic Agents	Fenretinide	5288209	BMP2
		Tretinoin	444795	BMP2
		Mitoxantrone	4212	EGR1
		Protopanaxadiol	9920281	EGR1
		Pyrazolanthrone	8515	EGR1
		Tephrosin	114909	NRG1
		Pterostilbene	5281727	NRG1
3	Antihypertensive Agents	Losartan	3961	BMP2
		Enalapril	5388962	EGR1
		Metoprolol	4171	EGR1
		Nimodipine	4497	EGR1
		Propranolol	4946	EGR1
		Reserpine	5770	EGR1
4	natural product	Aucubin	91458	BMP2
		Beta-glycerophosphoric acid	2526	BMP2
		Protopanaxadiol	9920281	EGR1
		Pterostilbene	5281727	NRG1
		Tephrosin	114909	NRG1
5	Anti-Arrhythmia Agents	Losartan	3961	BMP2
		Digoxin	2724385	EGR1
		Metoprolol	4171	EGR1
		Propranolol	4946	EGR1
5	Antioxidants	3,4-dihydroxyphenylethanol	82755	EGR1
		Resveratrol	445154	BMP2 EGR1 NRG1
		Pterostilbene	5281727	NRG1
7	Arsenicals	4-aminophenylarsenoxide	14290	MT1G MT1H MT1X
7	Protein Kinase Inhibitors	2-(2-amino-3-methoxyphenyl)-4H-1-benzopyran-4-one	4713	EGR1 NRG1
		Pyrazolanthrone	8515	EGR1
7	Platelet Aggregation Inhibitors	3,4-dihydroxyphenylethanol	82755	EGR1
		Resveratrol	445154	BMP2 EGR1 NRG1
		3-(4-methylphenylsulfonyl)-2-propenenitrile	5353431	EGR1
7	Vasodilator Agents	Colforsin	47936	EGR1
		Nimodipine	4497	EGR1
		Propranolol	4946	EGR1
7	Antipsychotic Agents	Reserpine	5770	EGR1

		Risperidone	5073	EGR1 NRG1
12	Hydroxymethylglutaryl-CoA Reductase Inhibitors	Atorvastatin	60823	BMP2
		Cerivastatin	446156	BMP2
12	Insecticides	Azadirachtin	5281303	BMP2
		1-naphthylisothiocyanate	11080	EGR1
12	Antineoplastic Agents, Phytogenic	Ginsenoside Rg3	9918693	BMP2
		Silybin	31553	EGR1
12	Histone Deacetylase Inhibitors	Trichostatin A	444732	BMP2 EGR1
12	Protein Synthesis Inhibitors	Trichostatin A	444732	BMP2 EGR1
12	Antifungal Agents	Trichostatin A	444732	BMP2 EGR1
12	Cardiotonic Agents	Colforsin	47936	EGR1
		Digoxin	2724385	EGR1
12	Neuroprotective Agents	Dizocilpine maleate	6420042	EGR1
		Pterostilbene	5281727	NRG1
12	Cardiovascular Agents	Indomethacin	3715	EGR1
		Ranolazine	56959	EGR1
12	Dopamine Antagonists	Risperidone	5073	EGR1 NRG1
12	Serotonin Antagonists	Risperidone	5073	EGR1 NRG1
12	Muscarinic Antagonists	Scopolamine	3000322	EGR1
		Propiverine	4942	HSD17B6
24	Carcinogenic	Glycidyl methacrylate	7837	MT1X
24	coating components	Glycidyl methacrylate	7837	MT1X
24	A gap junction inhibitor	18alpha-glycyrrhetinic acid	73398	BMP2
24	Herbicides	1-Methyl-4-phenylpyridinium (Cyperquat)	39484	BMP2
24	osteogenic activity	(4-(7-chloroquinolin-4-yl)piperazino)(1-phenyl-5-(trifluoromethyl)-1H-pyrazol-4-yl)methanone	310264838 (SID)	BMP2
24	Anticholesteremic Agents	Atorvastatin	60823	BMP2
24	Antimetabolites, Antineoplastic	Decitabine	451668	BMP2
24	Adrenergic alpha-Agonists	Epinephrine	5816	BMP2
24	Antineoplastic Agents, Hormonal	Tamoxifen	2733526	NRG1
24	Anticarcinogenic Agents	Fenretinide	5288209	BMP2
24	Angiotensin II Type 1 Receptor Blockers	Losartan	3961	BMP2
24	Hemostatics	Menatetrenone	5282367	BMP2
24	Hormones	Pasireotide	9941444	BMP2
24	Keratolytic Agents	Tretinoin	444795	BMP2
24	Chelating Agents	1,2-bis(2-aminophenoxy)ethane N,N,N',N'-tetraacetic acid acetoxymethyl ester	3034747	EGR1
24	Immunologic Factors	15-deoxy-delta(12,14)-prostaglandin J2	5311211	EGR1
24	Anilides	2-chloro-5-nitrobenzanilide (GW9662)	644213	EGR1
24	Anti-Infective Agents	3,4-dihydroxyphenylethanol	82755	EGR1
24	inhibitor of cytokine-induced IkappaB-alpha phosphorylation	3-(4-methylphenylsulfonyl)-2-propenenitrile	5353431	EGR1
24	Prostaglandin Antagonists	6-isopropoxy-9-oxoxanthene-2-carboxylic acid	119461	EGR1
24	Adjuvants, Immunologic	Colforsin	47936	EGR1
24	Bronchodilator Agents	Colforsin	47936	EGR1
24	Excitatory Amino Acid Antagonists	Dizocilpine maleate	6420042	EGR1
24	Hallucinogens	Dronabinol	16078	EGR1
24	Psychotropic Drugs	Dronabinol	16078	EGR1
24	Cannabinoid Receptor Agonists	Dronabinol	16078	EGR1
24	Analgesics, Non-Narcotic	Dronabinol	16078	EGR1

24	Angiotensin-Converting Enzyme Inhibitors	Enalapril	5388962	EGR1
24	Anti-Inflammatory Agents, Non-Steroidal	Indomethacin	3715	EGR1
24	Tocolytic Agents	Indomethacin	3715	EGR1
24	Cyclooxygenase Inhibitors	Indomethacin	3715	EGR1
24	Gout Suppressants	Indomethacin	3715	EGR1
24	Calcium Ionophores	Ionomycin	6912226	EGR1
24	Sympatholytics	Metoprolol	4171	EGR1
24	Adrenergic beta-1 Receptor Antagonists	Metoprolol	4171	EGR1
24	Cross-Linking Reagents	Mitomycin	5746	EGR1
24	Alkylating Agents	Mitomycin	5746	EGR1
24	Antibiotics, Antineoplastic	Mitomycin	5746	EGR1
24	Nucleic Acid Synthesis Inhibitors	Mitomycin	5746	EGR1
24	Topoisomerase II Inhibitors	Mitoxantrone	4212	EGR1
24	Analgesics	Mitoxantrone	4212	EGR1
24	Calcium Channel Blockers	Nimodipine	4497	EGR1
24	Adrenergic beta-Antagonists	Propranolol	4946	EGR1
24	Sodium Channel Blockers	Ranolazine	56959	EGR1
24	Antimitotic Agents	Razoxane	30623	EGR1
24	Adrenergic Uptake Inhibitors	Reserpine	5770	EGR1
24	Cholinergic Antagonists	Scopolamine	3000322	EGR1
24	Mydriatics	Scopolamine	3000322	EGR1
24	Adjuvants, Anesthesia	Scopolamine	3000322	EGR1
24	Antiemetics	Scopolamine	3000322	EGR1
24	Protective Agents	Silybin	31553	EGR1
24	Contraceptive Agents, Hormonal	Sulprostone	5312153	EGR1
24	Abortifacient Agents, Nonsteroidal	Sulprostone	5312153	EGR1
24	Menstruation-Inducing Agents	Sulprostone	5312153	EGR1
24	Anesthetics, Inhalation	Trichloroethylene (TCE)	6575	EGR1
24	Solvents	Trichloroethylene (TCE)	6575	EGR1
24	Nicotinic Antagonists	Vecuronium bromide	39764	EGR1
24	Neuromuscular Nondepolarizing Agents	Vecuronium bromide	39764	EGR1
24	Androgens	Dihydrotestosterone	10635	HSD17B6
24	Urological Agents	Propiverine	4942	HSD17B6
24	Parasympatholytics	Propiverine	4942	HSD17B6
24	Antiperspirant	Aluminum chlorhydrate	406845004 (SID)	NRG1
24	Mutagens	Aristolochic acid I	2236	NRG1
24	Carcinogens	Aristolochic acid I	2236	NRG1
24	Bone Density Conservation Agents	Tamoxifen	2733526	NRG1
24	Estrogen Antagonists	Tamoxifen	2733526	NRG1
24	Selective Estrogen Receptor Modulators	Tamoxifen	2733526	NRG1
24	pesticidal activities	Tephrosin	114909	NRG1
