# Supplementary material for: A System Biology Approach Reveals New Targets for Human Thyroid Gland Toxicity in Embryos and Adult Individuals
Source: Metabolites. 2024 Apr 16;14(4):226. doi: 10.3390/metabo14040226 (PMC11052307; doi:10.3390/metabo14040226)
Supplement: Supplementary file 1 [file metabolites-14-00226-s001.zip › metabolites-2942682-SI/Suppl Table S4 _ Down AT_CTD.pdf]

Supplementary Table S4 - Summary of the interaction between chemical compounds and downregulated genes in adult thyroid

Rank	MeSH Pharmacological Classification	Chemical Compound	PubChem CID	Gene/Protein interaction
1	Antineoplastic Agents	Tretinoin	444795	DEFA3
		Neocuproine	65237	CXCL1
		Crizotinib	11626560	CDA
		Oxaliplatin	9887053	CDA<br>TYMS
		Bicalutamide	2375	TYMS
		Gefitinib	123631	TYMS
		Palbociclib	5330286	TYMS
		Pemetrexed	135410875	TYMS
		Vorinostat	5311	TYMS
		Pyrazolanthrone	8515	CXCL1
		Cinobufagin	11969542	TYMS
		Paclitaxel	36314	LGALS4<br>CDA<br>TYMS
1	Antimetabolites, Antineoplastic	S 1 (combination)	54715158	PRSS2<br>PRSS3<br>LGALS4<br>TYMS
		Capecitabine	60953	LGALS4<br>TYMS
		Doxifluridine	18343	LGALS4<br>TYMS
		Decitabine	451668	CDA
		Gemcitabine	60750	CDA<br>RRM2
		Cytarabine	6253	TYMS
		Floxuridine	5790	TYMS
		Nolatrexed	135400184	TYMS
		Raltitrexed	135400182	TYMS
3	Enzyme Inhibitors	Acetovanillone /apocynin	2214	CXCL1
		Metyrapone	4174	CXCL1
		Resveratrol	445154	CXCL1
		Decitabine	451668	CDA
		Cyclosporine	5284373	TYMS
		Nolatrexed	135400184	TYMS
		Pemetrexed	135410875	TYMS
		Fluorodeoxyuridylate	8642	TYMS
4	Anti-Inflammatory Agents, Non-Steroidal	Acetovanillone /apocynin	2214	CXCL1
		Indomethacin	3715	CXCL1
		Mesalamine	4075	CXCL1
		Shikonin	479503	CXCL1
		Sulfasalazine	483928815<br>(SID)	CXCL1
		Zileuton	60490	CXCL1
4	Carcinogens	Azoxymethane	33184	CXCL1
		Phorbol 12,13-dibutyrate	37783	CXCL1
		Aristolochic acid I	2236	RRM2
		Glycidyl methacrylate	7837	TYMS
		Sterigmatocystin	5280389	CXCL1
		Glycidyl methacrylate	7837	TYMS
6	Immunosuppressive Agents	Triptolide	107985	CXCL1
		Doxifluridine	18343	LGALS4<br>TYMS
		Cyclosporine	5284373	TYMS
		Cytarabine	6253	TYMS
		Doxifluridine	18343	LGALS4

				TYMS
7	Protein Kinase Inhibitors	Doramapimod	156422	CXCL1
		Pyrazolanthrone	8515	CXCL1
		Erlotinib hydrochloride	176871	TYMS
		Palbociclib	5330286	TYMS
7	Dermatologic Agents	Ferric oxide	53790672 (SID)	CXCL1
		Fluticasone	5311101	CXCL1
		Cyclosporine	5284373	TYMS
		Hesperidin	10621	CXCL1
9	Antioxidants	Acetovanillone /apocynin	2214	CXCL1
		Ascorbic acid	54670067	CXCL1
		Resveratrol	445154	CXCL1
9	Natural product	Astringin	5281712	CXCL1
		Coagulin-L	10508451	CXCL1
		Monocrotaline	9415	CXCL1
9	Anti-Inflammatory Agents	Belnacasan	11398092	CXCL1
		Budesonide	5281004	CXCL1
		Fluticasone	5311101	CXCL1
9	Antihypertensive Agents	Benazepril	5362124	CXCL1
		Candesartan	2541	CXCL1
		Losartan	3961	CXCL1
9	Anti-Allergic Agents	Cetirizine	2678	CXCL1
		Fluticasone	5311101	CXCL1
		Methapyrilene	4098	CXCL1
9	Tyrosine Kinase Inhibitors	Quizartinib	24889392	TYMS
		Crizotinib	11626560	CDA
		Gefitinib	123631	TYMS
9	Nucleic Acid Synthesis Inhibitors	Mitomycin	5746	CDA
		Idoxuridine	5905	TYMS
		Pemetrexed	135410875	TYMS
9	Antifungal Agents	Ciclopirox	2749	RRM2
		Cyclosporine	5284373	TYMS
		Trichostatin A	444732	TYMS
9	Antiviral Agents	Cytarabine	6253	TYMS
		Idoxuridine	5905	TYMS
		Stallimycin	3115	TYMS
9	Folic Acid Antagonists	Nolatrexed	135400184	TYMS
		Pemetrexed	135410875	TYMS
		Raltitrexed	135400182	TYMS
9	Reagents and solvents	Hydrochloric acid	313	CXCL1
		Mitomycin	5746	CDA
		Pseudocumene	7247	CXCL1
20	Muscarinic Antagonist	Atropine	174174	CXCL1
		Benztropine	1201549	TYMS
20	Bronchodilator Agents	Budesonide	5281004	CXCL1
		Fluticasone	5311101	CXCL1
20	Angiotensin II Type 1 Receptor Blockers	Candesartan	2541	CXCL1
		Losartan	3961	CXCL1
20	fragrance component	Diacetyl	650	CXCL1
		Undecane	14257	CXCL1
20	Histamine H2 Antagonists	Famotidine	5702160	CXCL1
		Ranitidine	3001055	CXCL1
20	Anti-Ulcer Agents	Famotidine	5702160	CXCL1
		Ranitidine	3001055	CXCL1
20	Anesthetics, Inhalation	Halothane	3562	CXCL1
		Sevoflurane	5206	CXCL1
20	Antimetabolites	Metyrapone	4174	CXCL1
		Tetrahydrouridine	29243	CDA
20	Platelet Aggregation Inhibitors	Resveratrol	445154	CXCL1
		Sevoflurane	5206	CXCL1
20	Anti-Infective Agents	Sulfasalazine	483928815 (SID)	CXCL1
		Trovafloxacin	62959	CXCL1

20	Antirheumatic Agents	Sulfasalazine	483928815 (SID)	CXCL1
		Cyclosporine	5284373	TYMS
20	Antineoplastic Agents, Alkylating	Triptolide	107985	CXCL1
		Melphalan	460612	CDA
20	Appetite Stimulants	Doxifluridine	18343	LGALS4 TYMS
20	Antiparasitic/ Pediculicide	Ivermectin	6321424	RRM2 TYMS
20	coating components	Glycidyl methacrylate	7837	TYMS
20	Histone Deacetylase Inhibitors	Trichostatin A	444732	TYMS
		Vorinostat	5311	TYMS
20	Cholinesterase Inhibitors	Isoflurophate	5936	CXCL1
		Pyridostigmine bromide	7550	CXCL1
37	Keratolytic Agents	Tretinoin	444795	DEFA3
37	Angiotensin-Converting Enzyme Inhibitors	Benazepril	5362124	CXCL1
37	Glucocorticoids	Budesonide	5281004	CXCL1
37	Flavouring Agents	Carvacrol	10364	CXCL1
37	Histamine H1 Antagonists, Non-Sedating	Cetirizine	2678	CXCL1
37	Chemical Warfare Agents	Chlorine	24526	CXCL1
37	anti-hyperglycemic agent	Coagulin-L	10508451	CXCL1
37	Insect Repellents	Deet	4284	CXCL1
37	Androgens	Dihydrotestosterone	10635	CXCL1
37	Cannabinoid Receptor Agonists	Dronabinol	16078	CXCL1
37	Analgesics, Non-Narcotic	Dronabinol	16078	CXCL1
37	Hallucinogens	Dronabinol	16078	CXCL1
37	Psychotropic Drugs	Dronabinol	16078	CXCL1
37	Free Radical Scavengers	Edaravone	4021	CXCL1
37	Neuroprotective Agents	Edaravone	4021	CXCL1
37	Calcium Channel Agonists	Icilin	161930	CXCL1
37	Sodium Channel Agonists	Icilin	161930	CXCL1
37	Tocolytic Agents	Indomethacin	3715	CXCL1
37	Cyclooxygenase Inhibitors	Indomethacin	3715	CXCL1
37	Gout Suppressants	Indomethacin	3715	CXCL1
37	Cardiovascular Agents	Indomethacin	3715	CXCL1
37	Protease Inhibitors	Isoflurophate	5936	CXCL1
37	Analgesics	Ketamine	3821	CXCL1
37	Anesthetics, Dissociative	Ketamine	3821	CXCL1
37	Excitatory Amino Acid Antagonists	Ketamine	3821	CXCL1
37	Anti-Arrhythmia Agents	Losartan	3961	CXCL1
37	Histamine H1 Antagonists	Methapyrilene	4098	CXCL1
37	Hypnotics and Sedatives	Methapyrilene	4098	CXCL1
37	Chelating Agents	Neocuproine	65237	CXCL1
37	Antibacterial	Polymyxin B	483926164 (SID)	CXCL1
37	Sphingosine 1 Phosphate Receptor Modulators	Siponimod	44599207	CXCL1
37	Gastrointestinal Agents	Sulfasalazine	483928815 (SID)	CXCL1
37	Air Pollutants	Sulfur dioxide	1119	CXCL1
37	Antispermatogenic Agents	Triptolide	107985	CXCL1
37	Antiemetics	Tropisetron	656665	CXCL1
37	Serotonin 5-HT3 Receptor Antagonists	Tropisetron	656665	CXCL1
37	Topoisomerase II Inhibitors	Trovafloxacin	62959	CXCL1
37	Lipoxygenase Inhibitors	Zileuton	60490	CXCL1
37	Leukotriene Antagonists	Zileuton	60490	CXCL1
37	Antipruritics	Menthol	1254	CXCL6
37	Myeloablative Agonists	Doxifluridine	18343	LGALS4 TYMS
37	Alkylating Agents	Melphalan	460612	CDA
37	Antibiotics, Antineoplastic	Mitomycin	5746	CDA

37	Mutagens	Mitomycin	5746	CDA
37	Topoisomerase I Inhibitors	Camptothecin	24360	RRM2
37	Antineoplastic Agents, Phytogenic	Camptothecin	24360	RRM2
37	Chemosterilants	Alpha-Chlorohydrin	7290	TYMS
37	Antiparkinson Agents	Benztropine	1201549	TYMS
37	Dopamine Uptake Inhibitors	Benztropine	1201549	TYMS
37	Parasympatholytics	Benztropine	1201549	TYMS
37	Androgen Antagonists	Bicalutamide	2375	TYMS
37	Calcineurin Inhibitors	Cyclosporine	5284373	TYMS
37	Antidotes	Leucovorin	135403648	TYMS
37	Protein Synthesis Inhibitors	Trichostatin A	444732	TYMS
37	Phenanthrenes	Boldine methine	152186869 (SID)	CXCL1
